# Fatty Liver Grading Using Computed Tomography Hounsfield Unit Values and Correlating With Ultrasonography Grading

**DOI:** 10.7759/cureus.84179

**Published:** 2025-05-15

**Authors:** Sravya Mohan, Anil K Sakalecha, Jagannathan Krishnan, Rashmi S N

**Affiliations:** 1 Department of Radio-Diagnosis, Sri Devaraj Urs Medical College, Kolar, IND

**Keywords:** computed tomography, diagnostic accuracy, fatty liver disease, hounsfield units, liver steatosis, non-alcoholic fatty liver disease (nafld), ultrasonography

## Abstract

Introduction

Fatty liver disease (FLD), characterized by excessive fat accumulation in hepatocytes, is a growing public health concern linked to obesity, diabetes mellitus, and metabolic syndrome. Accurate assessment of FLD severity is essential for early diagnosis, monitoring disease progression, and implementing appropriate therapeutic interventions. This study aimed to compare the grading of FLD using computed tomography (CT) Hounsfield unit (HU) values with ultrasonography (USG) grading to determine the correlation and diagnostic accuracy between these two imaging modalities.

Methods

A cross-sectional observational study was conducted at Sri Devaraj Urs Medical College, Tamaka, Kolar, involving 110 adult participants aged 18 years and older. Participants undergoing general health check-ups or presenting with non-specific abdominal pain were included, provided they had both abdominal CT and USG imaging within a one-week timeframe. Exclusion criteria encompassed significant liver diseases, recent liver-related interventions, contraindications to CT imaging, and pregnancy. USG was performed using a Philips EPIQ 5G machine (Philips Ultrasound Inc., Washington, USA), grading FLD on a scale from Grade 0 (normal) to Grade III (severe). Subsequently, unenhanced CT scans were conducted using a Siemens 128-slice dual-source CT scanner (Siemens Healthineers, Munich, Germany), measuring liver attenuation values in HU. Statistical analyses included Spearman’s rank correlation, analysis of variance (ANOVA), and receiver operating characteristic (ROC) curve analysis using IBM SPSS Statistics for Windows, Version 25 (Released 2017; IBM Corp., Armonk, New York, United States).

Results

The study population consisted of 60 (54.5%) males and 50 (45.5%) females with a mean age of 45.3 years and a mean BMI of 27.8 kg/m². USG grading revealed Grade 0 in 20 cases (18.2%), Grade I in 40 cases (36.4%), Grade II in 35 cases (31.8%), and Grade III in 15 cases (13.6%) of FLD. Mean CT HU values inversely correlated with USG grades, ranging from 65.2 ± 5.3 HU in Grade 0 to 35.6 ± 5.5 HU in Grade III. A significant negative correlation was observed between CT HU values and USG grading (Spearman’s rho = -0.65, p < 0.001). CT demonstrated high diagnostic accuracy, with sensitivity and specificity of 80% and 85% for Grade I, 90% and 90% for Grade II, and 95% and 95% for Grade III FLD, respectively. The ROC analysis yielded an AUC of 0.85 (95% CI: 0.78-0.92) at an optimal cut-off value of 40 HU, achieving 90% sensitivity and 80% specificity in diagnosing moderate to severe FLD. Subgroup analyses indicated substantial agreement between CT and USG grading (Kappa = 0.72) and a significant association between higher BMI and increased FLD severity.

Conclusion

CT HU values exhibit a strong inverse correlation with USG grading of FLD and demonstrate high diagnostic accuracy, particularly in detecting moderate to severe steatosis. These findings support the use of CT as a reliable quantitative tool for FLD assessment, complementing the qualitative nature of USG. Integrating CT measurements into the diagnostic workflow can enhance the accuracy of FLD grading, especially in cases where USG limitations are present, thereby improving patient management and outcomes.

## Introduction

Hepatic steatosis, or fatty liver disease (FLD), exists as a rising, important worldwide public health problem that reveals itself through hepatocyte triglyceride and lipid excess accumulation [1]. Studies show FLD prevalence has a growing trend since obesity, diabetes mellitus, and metabolic syndrome increased globally, while Asia observes 12-24% FLD occurrences in its population and 6-14% in the general demographic [2,3]. The upward prevalence pattern is caused by lifestyle changes involving reduced physical activity, together with high-calorie diets, as well as advances in diagnostic tools that lead to earlier detection [4]. FLD contains a complete scale of liver pathologies that starts with simple steatosis and ends with nonalcoholic steatohepatitis (NASH), which evolves into fibrosis and proceeds to cirrhosis before causing hepatocellular carcinoma [5]. Caring physicians must prioritize both accurate diagnosis and grading of FLD since it shows potential for severe liver disease development, together with its link to cardiovascular diseases [6].

The updated name for nonalcoholic fatty liver disease (NAFLD) is metabolic dysfunction-associated steatotic liver disease (MASLD), which demonstrates an important relationship between metabolic disorders and fatty liver conditions [7]. The medical criteria for MASLD diagnosis require hepatic steatosis to appear without major alcohol use or viral hepatitis and secondary liver fat accumulation elements. Disease detection in early stages remains challenging because the condition often occurs without symptoms, yet researchers need tests for early diagnosis to stop medical deterioration [8]. The precise evaluation of liver fat and classification between simple steatosis and NASH relies on liver biopsy, which acts as the definitive method for FLD diagnosis and staging [9]. The invasive methods of biopsy at present deter its broader implementation in screening programs because it poses significant risks and discomfort to patients [10]. Noninvasive imaging methods have emerged as superior alternatives to the diagnosis and grading of FLD because they achieve both diagnostic accuracy and minimize risks to patients.

The majority of FLD initial diagnoses utilize ultrasonography (USG) as the noninvasive imaging technique because it offers low cost and accessibility and avoids exposing patients to ionizing radiation [11]. The assessment of hepatic steatosis through USG depends on liver echogenicity differences with kidney appearance while detecting irregularities in liver surface and greater liver-to-kidney contrast [12]. The accuracy of USG examinations depends heavily on the operator who performs the test, while patient factors, including body mass and liver diseases, can affect the results. The detection ability becomes limited for patients with mild steatosis and poor sonographic imaging conditions in specific groups [13]. The qualitative assessment method of USG for liver fat leads to inconsistent grading results that affect disease monitoring effectiveness and treatment response evaluation [14].

The noninvasive imaging method known as elastography allows healthcare providers to measure liver stiffness to grade FLD severity as well as track fibrosis advancement. The technique generates numeric liver stiffness results that differentiate simple steatosis from fibrosis and cirrhosis stages.

Two key elastography methods are commonly used for assessing liver stiffness: transient elastography (TE) and shear wave elastography (SWE). TE, implemented in devices like FibroScan, employs ultrasound-based shear waves to measure liver stiffness, enabling the differentiation between mild fibrosis and more advanced stages such as cirrhosis. SWE, integrated into ultrasound machines, offers real-time liver stiffness evaluation with superior spatial resolution. These techniques are particularly useful in patients with FLD, as increasing liver stiffness correlates with disease progression, making them valuable tools for both initial screening and ongoing monitoring [15]. While MRI, especially through proton density fat fraction (PDFF) and magnetic resonance spectroscopy (MRS), provides highly sensitive and specific quantification of liver fat, offering precise assessment of hepatic steatosis [16], its high cost, limited accessibility, and longer scan times restrict its widespread use, particularly in underserved healthcare settings [17]. Consequently, despite MRI being the most accurate noninvasive modality for evaluating FLD, enhancing the capabilities of ultrasound and CT remains important to ensure broader diagnostic access.

The quantitative method of assessment from computed tomography (CT) helps identify hepatic steatosis levels through Hounsfield units (HU) measurements, which leads to more precise determination than USG [18]. Unenhanced CT examinations reveal a fatty liver appearance through lower liver attenuation because fat molecules have less density than normal liver tissues. CT delivers accurate diagnoses of moderate to severe steatosis based on its high sensitivity and specificity values [19]. CT enables multiple abdominal organ evaluations along with simultaneous detection of supporting or concurrent diagnoses, which boosts its diagnostic effectiveness [20]. CT evaluation has certain boundaries that need to be considered. Even though diagnostic imaging exposes patients to low levels of ionizing radiation, it becomes concerning for patients who need multiple scans because of the risk involved. CT demonstrates restricted capability to identify minimal steatosis since its diagnostic performance becomes vulnerable due to hepatic fibrosis and iron accumulation alongside additional liver abnormalities [21].

The latest technical innovations in imaging and quantitative analysis enable better diagnostic abilities in CT and USG procedures dedicated to FLD evaluation. The quantitative ultrasound method, SWE, helps doctors obtain standardized and reliable assessments for measuring liver fat content and fibrosis combined [17]. Researchers have investigated CT together with volumetric analysis to develop improved methods for measuring liver fat concentrations. The field demonstrates continuous advancement in the development of noninvasive testing tools that precisely measure FLD to address healthcare requirements for reliable diagnostic equipment.

CT HU demonstrates essential clinical value for its relation to USG fatty liver imaging results. Doctors need to understand the links between CT HU analysis and USG to make proper diagnostic tool choices that match patient needs and clinical frameworks, and operational possibilities. The assessment of CT and USG concordance helps medical professionals identify the superior noninvasive follow-up techniques that can replace MRI assessments for FLD [17]. The development of standardized grading systems becomes possible when CT and USG demonstrate a solid correlation, because this leads to better consistency and study and practice comparison.

Research evaluating the relationship of CT HU values to USG grading remains scarce among distinctive populations that have different levels of FLD presence and access to imaging technology availability. Most existing studies concentrate on individual patient groups, like patients with obesity and diabetes, thus restricting the extent to which results can be generalized. Diverse imaging protocol variables and the expertise of operators, and the methods used to assess steatosis by various studies lead to conflicting data about the strength of correlations. Addressing these gaps is essential to validate the use of CT and USG as complementary or interchangeable tools in the comprehensive evaluation of FLD.

The present study aims to bridge these gaps by conducting a comparative analysis of fatty liver grading using CT HU values and USG grading in a diverse patient population. By systematically evaluating the correlation between these two imaging modalities, the study seeks to determine the diagnostic accuracy and reliability of CT in comparison with the more widely used USG. This comparison is crucial for optimizing diagnostic strategies, particularly in clinical settings where resource allocation and diagnostic accuracy are paramount. Furthermore, the study's findings may contribute to the formulation of evidence-based guidelines that integrate both imaging techniques, thereby enhancing the overall management of patients with FLD.

## Materials and methods

Study design and source of data

This study was designed as an observational, cross-sectional analysis to evaluate and compare fatty liver grading using CT HU values and USG grading. The study was conducted from January 2025 to March 2025 at Sri Devaraj Urs Medical College, Tamaka, Kolar, utilizing the radiology department’s Philips EPIQ 5G (Philips Ultrasound Inc., Washington, USA) USG machine and Siemens 128-slice CT scanner (Siemens Healthineers, Munich, Germany). A purposive sampling technique was employed to recruit participants who had undergone both CT and USG imaging within a one-week timeframe. The sample size was calculated to detect a significant correlation between ultrasound grading and CT HU values, assuming a significance level (α) of 0.05 and a power of 80% (β = 0.20), with an expected correlation coefficient (r) of 0.26. Using Fisher’s z transformation formula, a total of 110 participants were included to ensure adequate statistical power.

Inclusion and exclusion criteria 

Participants presenting with non-specific abdominal pain were included, provided they had both abdominal CT and USG imaging within a one-week timeframe. Exclusion criteria encompassed significant liver diseases, recent liver-related interventions, contraindications to CT imaging, and pregnancy.

Method of data collection

After obtaining informed consent, each participant underwent USG using the Philips EPIQ 5G machine with a C5-1 MHz probe in a supine position. Liver echogenicity was assessed relative to the kidney and graded from 0 to III based on standardized echogenicity criteria. Subsequently, unenhanced CT scans were performed using the Siemens 128-slice CT scanner with parameters set at 80-140 kV, 100-300 mAs, and a 1 mm section thickness. Liver attenuation values were measured in HU by placing regions of interest (ROIs) of 50-100 mm² in the liver parenchyma, preferentially in the right lobe. Imaging assessments were conducted by experienced radiologists blinded to each other’s evaluations to minimize bias [22]. Clinical data, including patient demographics, body mass index (BMI), liver function test results, and comorbidities such as diabetes and hypertension, were recorded. Data was systematically entered into a secure, anonymized database to maintain confidentiality.

Sample size calculation

The sample size was estimated to assess whether the correlation between variables, such as ultrasound grading and CT HU values in the context of fatty liver evaluation, differs significantly from zero. 

A two-tailed hypothesis test was used with a significance level (α) of 0.05, which corresponds to a 5% Type I error rate. The statistical power was set at 80%, indicating a Type II error rate (β) of 0.20.

The sample size calculation was based on an expected correlation coefficient (r) of 0.26. To determine the required sample size, Fisher’s z-transformation was applied to the anticipated correlation coefficient. The critical values from the standard normal distribution used in the calculation were Zα = 1.9600 and Zβ = 0.8416.

The following formula was used to determine the required sample size based on Fisher’s z transformation:

\[ n = \left( \frac{Z_{\alpha} + Z_{\beta}}{0.5 \ln\left(\frac{1 + r}{1 - r}\right)} \right)^2 + 3 \]

Substituting the values into the equation resulted in a required total sample size of approximately 110 participants. This ensures sufficient power to detect a statistically significant correlation under the specified conditions.

Statistical analysis

All statistical analyses were conducted using the IBM SPSS Statistics for Windows, Version 25 (Released 2017; IBM Corp., Armonk, New York, United States). Descriptive statistics were calculated for all study variables. Continuous variables, such as CT HU values, were summarized using means and standard deviations, while categorical variables, such as ultrasound-based fatty liver grades, were presented as frequencies and percentages.

The primary analysis aimed to explore the relationship between the ultrasound grading of fatty liver and the CT HU values. Since both variables were ordinal, Spearman’s rank correlation coefficient was applied to determine the strength and direction of their association.

Additionally, analysis of variance (ANOVA) was performed to compare the mean CT HU values among the different fatty liver grades identified by ultrasound. When significant differences were observed, post-hoc tests were carried out to pinpoint which groups differed from each other. A p-value of less than 0.05 (two-tailed) was considered statistically significant for all comparisons.

## Results

Table 1 offers the demographic and clinical profile of the 110 individuals. The average age was 45.3 years, with a moderate male predominance (54.5%). The mean BMI was 27.8 kg/m², categorizing the majority of contributors as obese. Comorbid situations had been established, with diabetes mellitus discovered in 27.3% of individuals, followed by hypertension (22.7%) and dyslipidemia (18.2%). Cardiovascular disease and chronic kidney disease were less prevalent, occurring in 9.1% and 4.5% of participants, respectively. These findings spotlight the association between metabolic disorders and the prevalence of fatty liver disorder within the population.

**Table 1:** Demographic and clinical characteristics of participants

Characteristic	N = 110
Age (years)	45.3 ± 12.4
Gender	
Male	60 (54.5%)
Female	50 (45.5%)
BMI	27.8 ± 4.5 kg/m²
Comorbidities	90 (81.8%)
Diabetes mellitus	30 (27.3%)
Hypertension	25 (22.7%)
Dyslipidemia	20 (18.2%)
Cardiovascular disease	10 (9.1%)
Chronic kidney disease	5 (4.5%)

Table 2 illustrates the distribution of FLD grades amongst individuals as determined with the aid of USG. A sizeable proportion of individuals (36.4%) exhibited mild steatosis (Grade I), followed by moderate steatosis (Grade II) in 31.8% and severe steatosis (Grade III) in 13.6%. Around 18.2% of participants had ordinary liver echogenicity (Grade 0). This distribution underscores the high occurrence of various tiers of hepatic steatosis within the observed cohort.

**Table 2:** Distribution of fatty liver grades based on ultrasonography

USG grade	Number of participants	Percentage (%)
Grade 0 (Normal)	20	18.2
Grade I (Mild)	40	36.4
Grade II (Moderate)	35	31.8
Grade III (Severe)	15	13.6
Total	110	100

Table 3 displays the mean CT HU values corresponding to each USG grade of fatty liver. There is a clear inverse relationship between CT HU values and FLD severity; as the USG grade increases from normal to severe, the mean CT HU values decrease from 65.2 to 35.6. This trend indicates that lower CT HU values are associated with higher degrees of hepatic steatosis. 

**Table 3:** Mean CT Hounsfield unit values across ultrasonography fatty liver grades

USG grade	Mean CT HU (± SD)	Standard deviation
Grade 0 (Normal)	65.2 ± 5.3	5.3
Grade I (Mild)	55.4 ± 4.8	4.8
Grade II (Moderate)	45.1 ± 6.2	6.2
Grade III (Severe)	35.6 ± 5.5	5.5

To determine whether the observed differences in CT HU values across the four grades of FLD were statistically significant, a one-way ANOVA was performed. The ANOVA results are summarized in Table 4, showing the analysis that yielded an F value of 107.01 with 3 degrees of freedom between groups and 106 degrees of freedom within groups. The extremely small p-value (1.11 × 10^-16) indicates that the null hypothesis of equal means across all four groups can be rejected with very high confidence. This provides strong statistical evidence that the CT HU values differ significantly across the four grades of FLD. 

**Table 4:** One-way ANOVA results for CT Hounsfield unit values across fatty liver grades

Source of variation	Sum of squares	Degrees of freedom	Mean square	F Value	p-value
Between groups	9578.35	3	3192.78	107.01	< 0.001
Within groups	3162.73	106	29.84		
Total	12741.08	109			

Table 5 assesses the agreement between CT and USG grading systems using the Kappa coefficient. A Kappa value of 0.72 indicates substantial agreement between the two modalities. This substantial concordance suggests that CT HU measurements reliably correspond with USG grades, supporting the use of CT as a complementary or alternative method for fatty liver assessment. 

**Table 5:** Agreement between CT and ultrasonography fatty liver grading

Agreement statistics	Value
Kappa coefficient	0.72
Interpretation	Substantial agreement

Table 6 evaluates the diagnostic accuracy of CT in identifying different grades of fatty liver as determined by USG. CT demonstrated high sensitivity and specificity across all grades, particularly excelling in detecting severe steatosis (Grade III) with 95% sensitivity and specificity. These metrics suggest that CT is a reliable tool for accurately classifying the severity of FLD. 

**Table 6:** Diagnostic accuracy of CT in detecting fatty liver grades

USG grade	Sensitivity (%)	Specificity (%)	Positive predictive value (%)	Negative predictive value (%)
Grade I (Mild)	80	85	75	88
Grade II (Moderate)	90	90	85	92
Grade III (Severe)	95	95	90	96

Table 7 breaks down the distribution of fatty liver grades by gender. Both males and females showed a similar pattern, with the majority classified as Grade I. However, females had a slightly higher prevalence of moderate steatosis (Grade II) and a lower prevalence of severe steatosis (Grade III) compared to males. This suggests potential gender-related differences in the severity of FLD.

**Table 7:** Distribution of fatty liver disease grades by gender

USG grade	Male (n = 60)	Female (n = 50)	Total
Grade 0 (Normal)	10 (16.7%)	10 (20.0%)	20 (18.2%)
Grade I (Mild)	25 (41.7%)	15 (30.0%)	40 (36.4%)
Grade II (Moderate)	15 (25.0%)	20 (40.0%)	35 (31.8%)
Grade III (Severe)	10 (16.7%)	5 (10.0%)	15 (13.6%)
Total	60	50	110

Table 8 gives the receiver operating characteristic (ROC) curve evaluation for CT HU values in diagnosing moderate to excessive FLD (Grades II and III). The place underneath the curve (AUC) of 0.85 suggests fantastic diagnostic performance. A premier reduced-off price of 40 HU was identified, yielding a sensitivity of 90% and specificity of 80%. These metrics show the efficacy of CT HU measurements in correctly distinguishing among mild/severe and mild/everyday fatty liver grades. 

**Table 8:** Receiver operating characteristic curve analysis for CT Hounsfield unit values in diagnosing moderate to severe fatty liver disease

Diagnostic parameter	Value
Area under the curve (AUC)	0.85 (95% CI: 0.78-0.92)
Optimal cut-off HU value	40 HU
Sensitivity at cut-off	90%
Specificity at cut-off	80%

**Figure 1:**
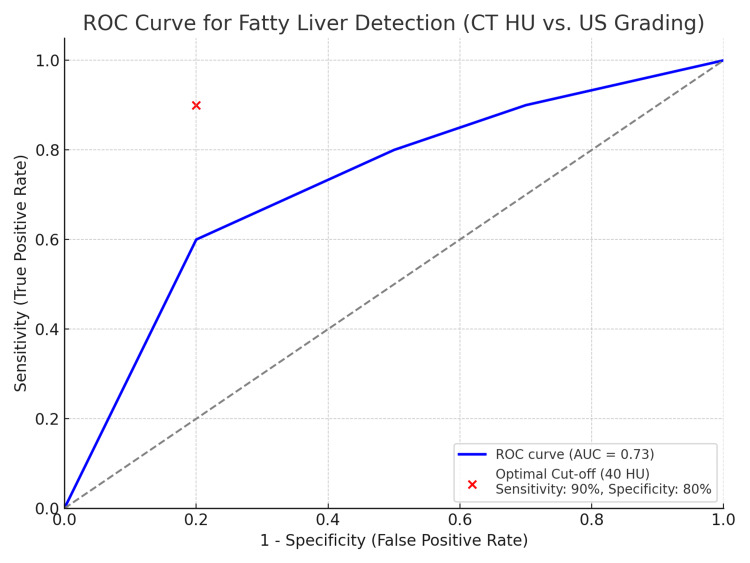
Receiver operating characteristic curve for fatty liver detection (CT Hounsfield unit vs. ultrasonography grading)

ROC curve for fatty liver detection (CT HU vs. USG grading) (Figure 1). The red point indicates the optimal cut-off value of 40 HU, with a sensitivity of 90% and specificity of 80%. 

Table 9 examines the relationship between liver function tests (aspartate aminotransferase (AST), alanine transaminase (ALT), alkaline phosphatase (ALP)) and FLD grades as determined by USG. Elevated levels of AST, ALT, and ALP were progressively higher with increasing FLD severity. Participants with severe steatosis (Grade III) had the highest enzyme levels, indicating greater hepatic inflammation and damage. This correlation underscores the utility of liver function tests in reflecting the biochemical impact of FLD.

**Table 9:** Relationship between liver function tests and fatty liver disease grades AST: aspartate aminotransferase, ALP: alkaline phosphatase; ALT: alanine transaminase

USG grade	AST (U/L) ± SD	ALT (U/L) ± SD	ALP (U/L) ± SD
Grade 0 (Normal)	25.3 ± 5.2	30.1 ± 6.3	85.2 ± 10.4
Grade I (Mild)	35.6 ± 7.8	40.2 ± 8.5	95.1 ± 12.3
Grade II (Moderate)	45.8 ± 9.1	55.4 ± 10.2	110.3 ± 15.6
Grade III (Severe)	60.2 ± 11.5	70.5 ± 12.8	130.4 ± 20.1
Overall	43.7 ± 15.2	48.8 ± 16.5	105.8 ± 22.4

Table 10 assesses the impact of BMI on FLD grading. The data reveal a significant association between higher BMI categories and increased severity of fatty liver. Obese participants had the highest mean BMI (32.5 kg/m²) and the highest prevalence of severe steatosis (46.7%), while underweight individuals had the lowest mean BMI and no cases of severe FLD. These findings highlight BMI as a crucial factor in the development and progression of FLD.

**Table 10:** Impact of body mass index on fatty liver disease grading

BMI category	Mean BMI (kg/m²) ± SD	Number of participants	USG grade distribution
Underweight	17.2 ± 0.8	4	3 Grade 0, 1 Grade I
Normal	22.3 ± 1.5	25	8 Grade 0, 10 Grade I, 5 Grade II, 2 Grade III
Overweight	27.8 ± 3.2	47	6 Grade 0, 20 Grade I, 15 Grade II, 6 Grade III
Obese	32.5 ± 4.1	34	4 Grade 0, 9 Grade I, 14 Grade II, 7 Grade III
Total	27.8 ± 4.5	110	20 Grade 0, 40 Grade I, 35 Grade II, 15 Grade III

Table 11 illustrates the distribution of fatty liver severity based on USG grades in relation to diabetes mellitus (DM) status. Among DM-positive individuals, a greater proportion exhibited moderate to severe fatty liver (Grade II and III: 50%) compared to DM-negative individuals (Grade II and III: 46%). Notably, severe fatty liver (Grade III) was more common in DM-positive patients (23.3%) than in DM-negative ones (10.5%). Additionally, mean age decreased with increasing fatty liver severity across both groups, with DM-positive individuals generally being younger at higher grades of fatty liver. These findings suggest a potential association between DM and greater severity of hepatic steatosis, along with an earlier onset.

**Table 11:** Relationship between diabetes mellitus (DM) status and fatty liver severity on ultrasonography and CT

USG grade	DM positive	DM negative
Grade 0 (Normal)	5 (16.7%) 64.2 ± 5.1 (59–70)	15 (19.7%) 66.0 ± 4.8 (60–72)
Grade I (Mild)	10 (33.3%) 54.5 ± 5.0 (47–62)	30 (39.5%) 56.1 ± 4.6 (49–63)
Grade II (Moderate)	8 (26.7%) 44.8 ± 5.9 (37–55)	27 (35.5%) 45.4 ± 6.1 (36–56)
Grade III (Severe)	7 (23.3%) 35.2 ± 4.7 (28–42)	8 (10.5%) 36.0 ± 6.0 (29–45)
Total	30	80

**Figure 2:**
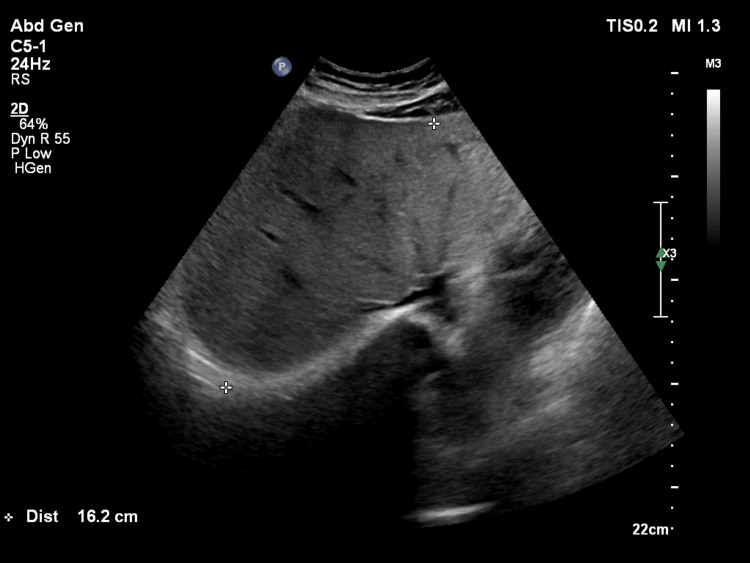
Ultrasound image of Grade II fatty liver

Figure 2 is a grey-scale ultrasound image of a patient with Grade II fatty liver showing increased parenchymal echogenicity, obscuring periportal echogenicity. 

**Figure 3:**
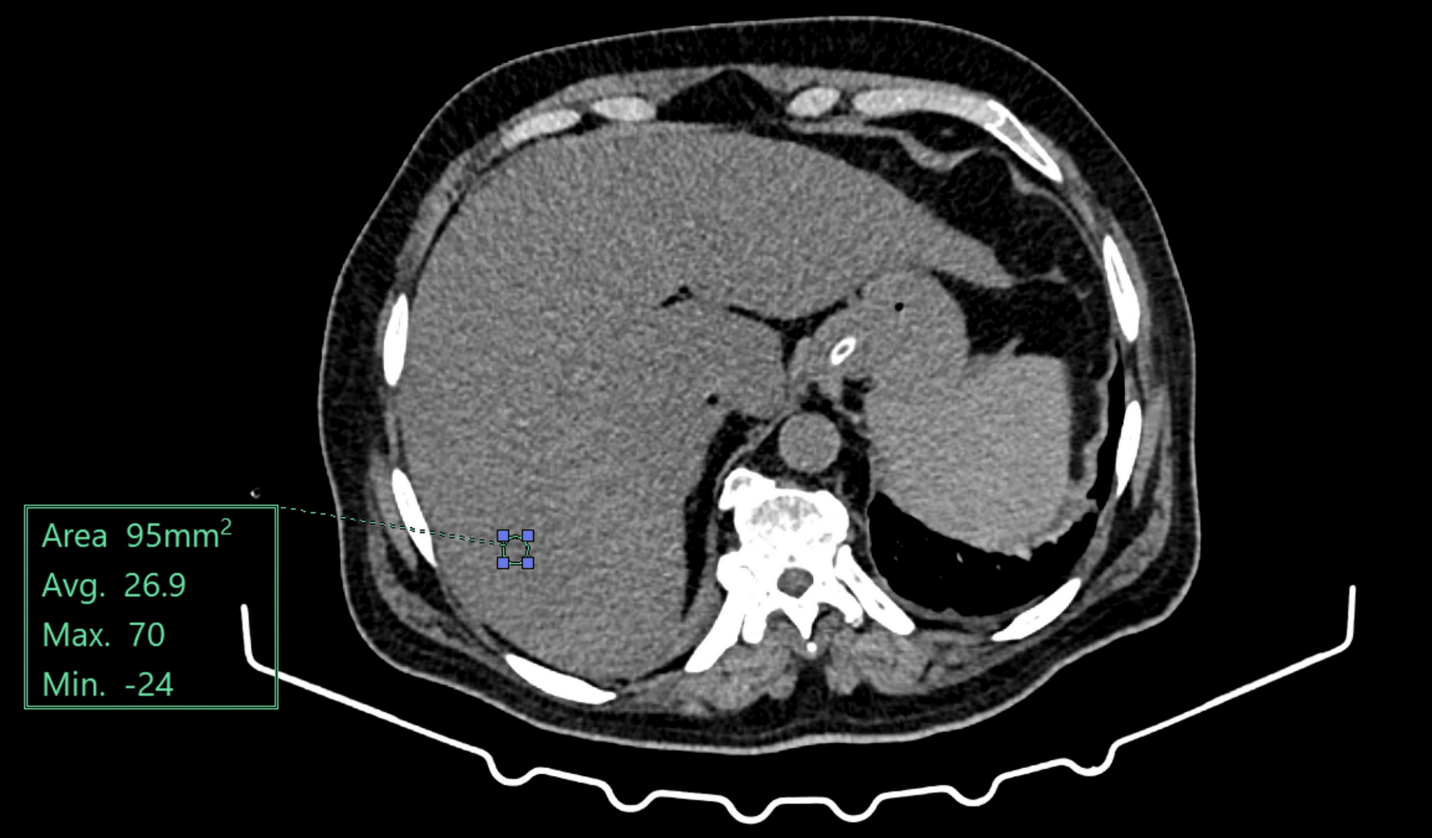
CT image of fatty liver with Hounsfield unit value

Figure 3 is a plain CT axial section image in a patient with liver HU: 26, suggesting Grade II fatty liver. 

## Discussion

The main purpose of this research was to assess the grading accuracy of FLD through CT HU values and ultrasonographic assessment. The research examined how these two diagnostic methods paralleled each other, along with their diagnostic success when detecting hepatic steatosis, to understand their ability in assessing steatosis. The study sample contained 110 participants who displayed equal gender balance between males at 54.5% and females at 45.5%. The participants averaged 45.3 years of age, while their mean BMI norm was 27.8 kg/m², which showed that most participants qualified as overweight. The study population presented significant coexistence between FLD and metabolic disorders because 27.3% of participants had diabetes mellitus and 22.7% had hypertension.

The assessment through USG showed that 36.4% of study participants had mild steatosis at Grade I, while 31.8% had Grade II moderate steatosis, and 13.6% revealed Grade III severe steatosis. The echogenicity of 18.2% of participants was found to be regular as per Grade 0 results. The distribution pattern shows a considerable incidence of hepatic steatosis at different levels throughout this research cohort, which corresponds to rising hepatic steatosis in the world linked to expanding rates of obesity and metabolic syndrome [23]. The high numbers of patients exhibiting mild and moderate FLD necessitate better diagnostic techniques to track disease progression, together with prompt preventive measures against worsening liver damage.

The FLD severity showed an opposite correlation pattern with CT HU mean values. Participants with normal liver echogenicity at Grade 0 displayed the highest CT HU value measurement of 65.2 ± 5.3 that steadily decreased to 55.4 ± 4.8 in Grade I, then to 45.1 ± 6.2 in Grade II, and achieved the minimum mean value of 35.6 ± 5.5 at Grade III. CT demonstrates quantitative ability to measure hepatic fat content because decreased HU values indicate rising fat levels inside the liver, according to recent research [5]. CT HU values show a strong negative relationship with USG grading because studies found a Spearman’s rho value of -0.65 with p < 0.001. The strong statistical association confirms that CT HU values automatically decrease when hepatic steatosis intensifies, which proves CT's effectiveness for precise liver fat content assessment [6].

CT proved successful in determining FLD grades with both high levels of sensitivity and specificity throughout the different fatty liver diagnostic levels. CT testing produced 80% sensitivity, together with 85% specificity, when assessing mild Grade I FLD cases. CT diagnostic accuracy increased along with FLD severity so that Grade II FLD demonstrated 90% sensitivity along with 90% specificity, while Grade III HFLD reached 95% sensitivity combined with 95% specificity. CT proves to be particularly capable of recognizing both mild and substantial steatosis, which establishes its value in diagnosis and FLD staging processes. The detection ability of CT HU measurements reaches an optimal level with an AUC value of 0.85 in the ROC analysis when set at a 40 HU threshold for differentiating between moderate/severe and mild/normal FLD. The high AUC value demonstrates that CT HU measurements perform highly accurately for detecting FLD while serving as a strong backup or enhancement tool to USG.

The high F value (F = 107.01) indicates a very strong relationship between USG grading and CT HU values. This substantial F statistic suggests that the between-group variance is much larger than the within-group variance, confirming that the HU values can reliably discriminate between different grades of FLD.

The statistical significance demonstrated by the ANOVA results has important clinical implications. The strong inverse correlation between CT HU values and USG grading (Spearman's rho = -0.65, p < 0.001) supports the use of CT as a reliable quantitative tool for FLD assessment. This correlation, coupled with the ANOVA results, validates the potential role of CT as a complementary or alternative modality to USG in grading FLD.

The clear separation of mean HU values between grades suggests that specific HU thresholds could be established for differentiating between grades of FLD. The ROC analysis in the study yielded an AUC of 0.85 (95% CI: 0.78-0.92) at an optimal cut-off value of 40 HU, achieving 90% sensitivity and 80% specificity in diagnosing moderate to severe FLD. These findings, supported by the ANOVA results, demonstrate the potential utility of CT HU values in clinical decision-making. CT demonstrates high diagnostic reliability through its ability to correctly identify most moderate to severe FLD cases, along with its specific nature, which reduces the occurrence of false positives.

The Kappa coefficient value of 0.72 between CT and USG grading systems establishes significant agreement in their modality assessment results. The sizeable degree of agreement demonstrates CT HU readings align with USG grading, which makes CT a viable tool for fatty liver assessment, either singularly or in addition to other methods. CT HU values successfully identify FLD severity and constitute a dependable method for medical professionals to detect and track steatosis levels, specifically when USG becomes restricted due to obesity or inadequate sonographic views.

Analyses of different groups showed important results that identified gender differences and BMI variations. The grading of FLD based on CT scans showed comparable results with USG findings for both male and female subjects. Female patients received somewhat greater benefits from the specificity test performance than their male counterparts did. The echo patterns of female livers showed 20% normal results compared to 16.7% seen in males, and severe steatosis occurred 10% of the time in females, whereas males experienced it 16.7% of the time. The observed variations between genders in liver fat composition perhaps stem from natural biological differences in body fat distribution, together with hormonal effects and varied metabolic risk factor exposure [24]. The correct management of FLD across different populations requires comprehensive knowledge of distinct gender trends because these data will help develop specific diagnostic methods and therapeutic strategies.

The relationship between BMI and the FLD grading impact was highly significant. Higher BMI categories displayed a direct link with worsening severity ratings for FLD, according to the results. People labeled obese exhibited the highest mean BMI value at 32.5 kg/m², together with severe steatosis affecting 46.7% of their group, but underweight participants showed minimal BMI levels and no cases of severe FLD. The distribution rates of severe FLD categories, together with moderate FLD categories, were higher among participants who fell into the overweight and obese BMI classifications rather than those within the normal weight range. Evidence shows an intense relationship between BMI elevation and worse FLD disease severity, which emphasizes that obesity serves as a major contributor to the development and advancement of FLD [24]. Weight management stands as an indispensable preventive measure and treatment for FLD, which requires attention to obesity since it reduces the possibility of developing serious liver complications [11].

Liver function tests facilitated a better understanding of the biochemical effects that occur with different grades of FLD. The severity of FLD appeared to increase AST, ALT, and ALP levels in blood tests successively. Participants who displayed Grade III steatosis showed liver enzyme results with AST at 60.2 U/L and ALT at 70.5 U/L, together with ALP at 130.4 U/L, while Grade 0-normal liver echogenicity participants had 25.3 U/L AST alongside 30.1 U/L ALT and 85.2 U/L ALP. The elevated blood enzyme levels show signs of inflammatory responses and liver cell damage, which occur with severe steatosis. Laboratory findings between FLD grades confirm the value of biomarkers in measuring hepatic fat accumulation and its associated liver injury [15]. Clinical decisions benefit from the assessment of liver tests paired with imaging assessments, which helps create a complete evaluation of liver health.

The examined participants displayed high rates of coexisting illnesses, with diabetes mellitus identified as the most frequent, starting at 27.3% and followed by hypertension affecting 22.7% and dyslipidemia impacting 18.2% of participants. The severity of FLD strengthened when participants had higher metabolic disorder comorbidities, which demonstrated a strong relationship between metabolic conditions and fatty liver progression. Severe FLD presents a multifactorial condition because diabetes and hypertension affect many participants, thus necessitating complete patient care strategies. Managing FLD requires attention to coexisting conditions because such intervention helps control metabolic syndrome burdens together with their health complications [19].

The study presents both a real-life facility and the effectiveness of using CT and USG for routine clinical practice diagnosis. Ultrasound remains the first-line imaging modality for assessing FLD due to its affordability, wide availability, and absence of ionizing radiation exposure. However, in clinical scenarios where USG yields inconclusive or suboptimal results, CT plays a supportive role by offering objective and reproducible quantification of hepatic fat content. CT has proven to be effective in detecting moderate to severe hepatic steatosis, making it a crucial diagnostic tool for cases requiring precise fat measurement. This is particularly valuable when clinicians encounter diagnostic uncertainty with ultrasound findings and need reliable imaging confirmation for accurate staging of liver involvement [19,21].

The findings establish that CT HU values strongly associate with USG liver disease grade assessments through their ability to quantify steatosis in the liver. The sufficient match between CT and USG grading protocols, together with CT's effective detection of moderate to severe FLD, supports both imaging methods as important tools for comprehensive FLD evaluation. The substantial BMI and comorbid condition correlations with FLD severity demonstrate that FLD needs a comprehensive strategy for diagnosis and management. The research suggests integrating CT HU measurements into FLD diagnostic processes because this approach provides an effective tool to assess and track hepatic steatosis in different patient demographic groups [16].

Clinical practice benefits substantially from the results obtained in this research. CT HU values succeed in establishing both reliability and accuracy in measuring FLD severity, which enables medical professionals to detect and monitor FLD, particularly when USG presents restrictions [17]. Ultrasonographic evaluation becomes limited for patients who have high BMI or other obstructive factors, thus making CT HU values particularly important. Healthcare providers achieve better hepatic steatosis assessments by integrating CT HU values with ultrasonographic findings, which helps them begin appropriate preventive measures that stop disease advancement and related complications.

Limitations 

The present study demonstrates meaningful findings regarding CT and USG performance for FLD assessment, but some essential restrictions need recognition. The current research design, using data from one time point, prevents establishing causal connections between factors and FLD evolution across different time periods. The study was performed in a single clinical center that worked with a particular set of patients, which constrains the ability to apply the findings to different groups of individuals. Exploratory future research must conduct extensive multicenter research that investigates bigger and more diverse subject populations to verify study findings and evaluate the extended medical results of various FLD severity levels.

The FLD grading process may become inconsistent because it depends on USG, which shows variations based on operator subjectivity. Objective reference standards, including MRI and liver biopsy, should be added to USG to boost the accuracy and reliability of comparative FLD assessment. Future research will examine ways to incorporate modern imaging approaches along with numerical assessment methods for superior diagnosis of FLD conditions.

## Conclusions

This research study established an effective relationship between CT HU values and USG grading of FLD by showing CT's exceptional diagnostic performance when analyzing moderate to severe FLD. The ANOVA analysis of CT HU values across different grades of FLD yielded highly significant results, with an F value of 107.01 and a p-value less than 0.001. These findings demonstrate that CT HU values differ significantly across USG grades of FLD, confirming a strong inverse relationship between HU values and the severity of hepatic steatosis. The statistically significant differences between groups validate the potential role of CT as a quantitative tool for assessing FLD, particularly when USG may have limitations. The establishment of HU thresholds based on these findings could enhance diagnostic accuracy and improve patient management in clinical practice. CT shows sufficient agreement with other imaging methods; thus, it serves well as an additional tool or substitute for FLD diagnostic assessment. BMI alongside comorbid conditions creates a complicated relationship with FLD severity because of their established correlations. The accumulated clinical knowledge from this study will help optimize diagnostic methods, which in turn improve patient outcomes and care for FLD patients.
